# Identification of Four Potential Biomarkers Associated With Coronary Artery Disease in Non-diabetic Patients by Gene Co-expression Network Analysis

**DOI:** 10.3389/fgene.2020.00542

**Published:** 2020-06-24

**Authors:** Min Jiao, Jingtian Li, Quan Zhang, Xiufeng Xu, Ruidong Li, Peikang Dong, Chun Meng, Yi Li, Lijuan Wang, Wanpeng Qi, Kai Kang, Hongjie Wang, Tao Wang

**Affiliations:** ^1^Department of Cardiology, Affiliated Hospital of Weifang Medical University, Weifang, China; ^2^Department of Neurology, Affiliated Hospital of Weifang Medical University, Weifang, China; ^3^Graduate Program in Genetics, Genomics, and Bioinformatics, University of California, Riverside, Riverside, CA, United States; ^4^Division of Cardiology, Department of Internal Medicine, Tongji Hospital, Tongji Medical College, Huazhong University of Science and Technology, Wuhan, China

**Keywords:** coronary artery disease, non-diabetic patients, weighted gene co-expression network analysis, function enrichment analysis, biomarkers

## Abstract

**Background:**

Coronary artery disease (CAD) is a type of cardiovascular disease that greatly hurts the health of human beings. Diabetic status is one of the largest clinical factors affecting CAD-associated gene expression changes. Most of the studies focus on diabetic patients, whereas few have been done for non-diabetic patients. Since the pathophysiological processes may vary among these patients, we cannot simply follow the standard based on the data from diabetic patients. Therefore, the prognostic and predictive diagnostic biomarkers for CAD in non-diabetic patient need to be fully recognized.

**Materials and Methods:**

To screen out candidate genes associated with CAD in non-diabetic patients, weighted gene co-expression network analysis (WGCNA) was constructed to conduct an analysis of microarray expression profiling in patients with CAD. First, the microarray data GSE20680 and GSE20681 were downloaded from NCBI. We constructed co-expression modules *via* WGCNA after excluding the diabetic patients. As a result, 18 co-expression modules were screened out, including 1,225 differentially expressed genes (DEGs) that were obtained from 152 patients (luminal stenosis ≥50% in at least one major vessel) and 170 patients (stenosis of <50%). Subsequently, a Pearson’s correlation analysis was conducted between the modules and clinical traits. Then, a functional enrichment analysis was conducted, and we used gene network analysis to reveal hub genes. Last, we validated the hub genes with peripheral blood samples in an independent patient cohort using RT-qPCR.

**Results:**

The results showed that the midnight blue module and the yellow module played vital roles in the pathogenesis of CAD in non-diabetic patients. Additionally, CD40, F11R, TNRC18, and calcium/calmodulin-dependent protein kinase type II gamma (CAMK2G) were screened out and validated using enzyme-linked immunosorbent assay (ELISA) in an independent patient cohort and immunohistochemical (IHC) staining in an atherosclerosis mouse model.

**Conclusion:**

Our findings demonstrate that hub genes, CD40, F11R, TNRC18, and CAMK2G, are surrogate diagnostic biomarkers and/or therapeutic targets for CAD in non-diabetic patients and require deeper validation.

## Introduction

Coronary artery disease (CAD), which is also called atherosclerotic cardiovascular disease and coronary heart disease (CHD), is the leading cause of cardiovascular deaths (CVDs) globally (Roth et al., 2017), with a death rate that is predicted to increase. The estimated number of deaths from CVD in 2008 was 17.3 million, comprising almost 30% of all deaths. Further, global CVDs are projected to increase to 23.4 million, comprising 35% of all deaths in 2030 (Ohira and Iso, 2013; Benjamin et al., 2019). CAD can cause vessel stenosis, which causes ischemia and atherosclerotic plaques that can rupture with the development of disease, which is the major mechanism for the occurrence of acute myocardial infarction (AMI). Furthermore, severely stenotic CAD, myocardial infarction, or chronic ischemia may cause heart failure and/or death (Thygesen et al., 2012). The treatment of CAD alleviates its manifestations and prevents the occurrence of AMI or premature death. Percutaneous coronary intervention (PCI) with coronary stenting and coronary artery bypass grafting (CABG) are widely used to reestablish adequate blood supply to ischemic myocardial areas, except for medical therapy to control the onset of angina (Neumann et al., 2019). Although scientists have worked hard to prevent and cure CAD and have made great progress, they are still faced with many challenges. It is urgent to study the mechanisms and a more effective therapeutic schedule for CAD. Recently, an increasing number of hub genes of CAD have been identified, providing further insight to comprehend the underlying molecular mechanisms. For instance, the research has revealed that TNPO1, RAP1B, ZDHHC17, and PPM1B might exert an important role in the occurrence and development of atherosclerosis (Zhang et al., 2019). Since diabetic status is one of the largest clinical factors affecting CAD-associated gene expression changes, most studies have focused on diabetic patients (Wingrove et al., 2008; Guo et al., 2018), and few have been done on non-diabetic patients (Sinnaeve et al., 2009; Elashoff et al., 2011). Additionally, since the pathophysiological processes may vary among these patients, we cannot simply follow the standard based on the data from diabetic patients. Therefore, to gain more insight into the latent molecular mechanisms of CAD in non-diabetic patients, the weighted gene co-expression network analysis (WGCNA) method was utilized in current research.

Weighted gene co-expression network analysis, which is a comprehensive collection of R functions, is a method of computational systems biology that is utilized to describe the pattern of gene correlation between phenotypic traits. We can find clusters (modules) of highly correlated genes using WGCNA. The hub genes of modules, described as the most closely associated with disease, often have more biological significance compared with the other genes of global networks (Goh et al., 2007; Wang et al., 2017). We can use WGCNA to identify the specific modules and hub genes that are correlated with phenotypes (Zhang and Horvath, 2005) and then to explore candidate biomarkers or therapeutic targets. Recently, WGCNA has been comprehensively applied in multiple diseases, such as breast cancer (Clarke et al., 2013), schizophrenia (de Jong et al., 2012), idiopathic pulmonary arterial hypertension (Wang et al., 2019), acute aortic dissection (Wang et al., 2017), and intracranial aneurysm (Zheng et al., 2015). Compared with the traditional microarray that is based on a microarray expression profiling data analysis, WGCNA takes the interaction of the transcriptome into account *via* constructing co-expression modules. Therefore, WGCNA makes the study more meaningful.

In our study, we first identified differentially expressed genes (DEGs) using WGCNA analysis and then constructed co-expression modules, pathway action network, and protein–protein interaction (PPI) networks to identify the pathways and hub genes. The hub genes CD40, F11R, TNRC18, and calcium/calmodulin-dependent protein kinase type II gamma (CAMK2G) were recognized as the most pivotal genes in the pathogenesis of CAD and could serve as biomarkers to diagnose CAD and be used as target genes to develop effective therapeutic schemes for CAD in non-diabetic patients.

## Materials and Methods

### Ethics Statement and Specimen Collection

All research protocols were approved by the Ethics Committee of the Affiliated Hospital of Weifang Medical University, Weifang, China. All of the patients or their relatives signed a written informed consent in conformity with the Declaration of Helsinki. The diagnosis of CAD patients was conducted by detecting flow-lowering in coronary artery stenoses by quantitative coronary angiography (QCA). The inclusion criterion for the CAD patient population Case (2) was coronary artery stenosis of ≥50% in at least one major coronary artery. The inclusion criterion for the control population Case (1) was luminal stenosis of less than 50% or no angiographically detectable coronary artery stenosis. As is well known, CAD is a kind of complex disease, and age, sex, CHD family history, smoking history, hypertension, diabetes, abnormal lipid metabolism, and insulin resistance are considered risk factors for CAD. Hypertension (especially uncontrolled hypertension) is the main risk factor for stroke, CHD, and all-cause mortality. In addition, end-organ damage, such as chronic kidney disease, is known to be an independent risk factor for cardiovascular diseases. Therefore, in order to eliminate the influence of confounding factors, exclusion criteria for Case (1) and Case (2) were diabetes, uncontrolled hypertension (systolic blood pressure >180 mmHg or diastolic blood pressure >100 mmHg), or end-organ damage (Sinnaeve et al., 2009). The characteristics of Case (1) and Case (2) samples for validation were shown in Table 1.

**TABLE 1:** Characteristics of patients and controls.

Clinical factor	Case (1) (*N* = 40)	Case (2) (*N* = 60)
Age (years)	54.6 ± 6.3	66.7 ± 5.8
Men (%)	68	73
**Medical history**		
Smoking (%)	42	48
Hypertension (%)	54	76
Diabetes Mellitus (%)	0	0
**Cardiovascular history**		
Unstable angina, %	0	60
Prior myocardial infarction, %	0	25
Prior percutaneous coronary intervention, %	0	15
Prior coronary artery bypass grafting, %	0	5
Congestive heart failure, %	0	0
Arrhythmia, predominantly atrial fibrillation, %	12.5	13.3
**Medications**		
Aspirin, %	40	93
ACE inhibitor, (%)	32	42
ARB, (%)	0	7
Beta blocker, (%)	25	67
Calcium blocker, (%)	12.5	21.6
Statin, (%)	15	75

*Data are presented as mean ± SD or (%).*

### Data Resources

**FIGURE 1:**
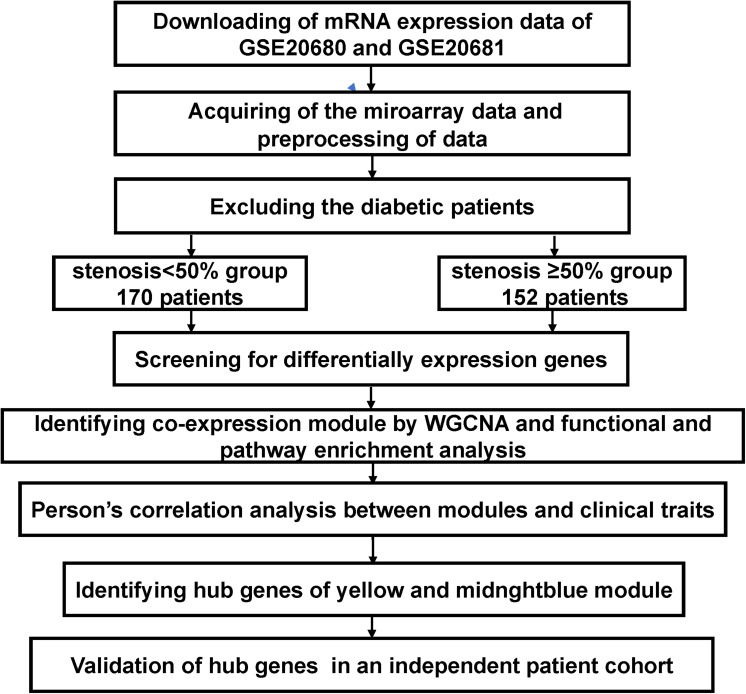
Flowchart of the analytical process in the current research: Data collection, preconditioning, data analysis, and validation. The mRNA microarray analysis procedures were performed on peripheral blood mononuclear cells (PBMCs) that were acquired from 152 coronary artery disease (CAD) patients with stenosis of coronary artery ≥50% and 170 patients in the control group with stenosis of coronary artery <50% in GSE20680 and GSE20681 after excluding the diabetic patients. Then, differentially expression genes (DEGs) were screened using the Mann–Whitney test analysis, and the yellow module and the midnight blue module were identified through weighted gene co-expression network analysis (WGCNA). Gene Ontology (GO) functional and Kyoto Encyclopedia of Genes and Genomes (KEGG) pathway enrichment analyses were performed in two significant modules. The four hub genes CD40, CAMK2G, F11R, TNRC18 were recognized and validated in an independent patient cohort using RT-qPCR and ELISA.

Figure 1 shows a flowchart of this research. The microarray dataset of CAD was downloaded from the Gene Expression Omnibus (GEO) database of NCBI^1^. We redivided it into two conditions after excluding the diabetic patients: Case (1) were patients with luminal stenoses of less than 50%, and Case (2) were patients with more than 50% stenosis in at least one major vessel by QCA. The two datasets GSE20680 (*n* = 124; ≥50%, 53; <50%, 71) (Elashoff et al., 2011) and GSE20681 (*n* = 198; ≥50%, 99; <50%, 99) (Beineke et al., 2012) were selected for analysis in the current study.

### Microarray Data Preprocessing

Raw data in GSE20686 (SuperSeries of GSE20680 and GSE20681) were downloaded from NCBI GEO by using R/Bioconductor package GEOquery (version 2.50.5). The platform was Agilent-014850 Whole Human Genome Microarray 4 × 44K G4112F (Agilent Technologies, Inc., Los Angeles, CA, United States). R/Bioconductor package Agi4 × 44PreProcess (version 1.22.0) was used for data normalization.

### Differentially Expressed Genes Screening

Mann–Whitney test analysis was used to identify the DEGs in Case (1) patients with luminal stenoses of <50% and Case (2) patients with ≥50% stenosis in at least one major vessel. Genes of *P* < 0.05 was performed as the threshold for the significant DEGs to analyze.

### Construction of Gene Co-expression Networks and Identification of Modules

Weighted gene co-expression network analysis is a typical systematic biological method that uses the soft thresholding to construct scale-free gene co-expression networks (Horvath and Dong, 2008). A Pearson’s correlation matrix was calculated for each set of genes to construct a gene co-expression network through counting a pairwise correlation matrix. Then, the Pearson correlation matrix was switched to a weighted adjacency matrix with a soft threshold power (β) set at 7 (scale-free *R*^2^ > 0.85). Next, using the blockwise modules function of the WGCNA package (version 1.68), we made the weighed adjacency matrix convert into a topological overlap matrix (TOM) (Ponsuksili et al., 2013). To group genes with highly similar expression patterns into gene co-expression modules, we established hierarchical average lineage clustering based on the TOM. In our study, the Pearson’s correlation between the module eigengene (ME) of each module and clinical information was defined as module significance (MS). During the selected modules, a module with the highest MS is defined as the one that has a high association with diseases. Then, the relationships during the feature vector of the module and the clinical traits were calculated to recognize the most relevant module (Wang et al., 2017, 2019).

### Gene Ontology Function and Kyoto Encyclopedia of Genes and Genomes Pathway Enrichment Analyses

Gene Ontology (GO) functional (updated in September 30, 2017) (Gene Ontology, 2015) and Kyoto Encyclopedia of Genes and Genomes (KEGG) pathway enrichment analyses (updated in March 31, 2017) (Kanehisa et al., 2016) were utilized in the yellow module and the midnight blue module (Figures 4, 5). The GO analysis described gene functions from three categories: biological process (BP), cellular component (CC), and molecular function (MF) (Tweedie et al., 2009). The KEGG pathway enrichment analysis is utilized to determine the significant pathway terms of molecules or genes (Kanehisa and Goto, 2000). Then, we calculated the *P*-value of each term using Fisher’s exact test, while *P* < 0.05 was considered significant.

### Protein–Protein Interaction Network Analysis and Hub Gene Identification

In this study, the genes in the yellow module and the midnight blue module were analyzed separately. PPI networks were constructed by the WGCNA analysis and the STRING database (version 10.5^2^), and the cutoff criterion for STRING database was set as a combined score >0.4 (the default parameter).

Specifically, the correlation of genes in the yellow module and the midnight blue module was obtained through WGCNA analysis. The correlation intensity is measured by weight. The weight value of genes ≥0.1 was considered to have a strong correlation. The gene network of the genes in the yellow module and the midnight blue module was generated by this correlation and visualized by Cytoscape (version 3.7.1 for Windows^3^) (Wan et al., 2018).

For hub gene identification, the number of genes with a strong correlation for each gene was counted, and the number of correlated genes in each model was more than 15 genes, which was considered a hub gene.

### Total RNA Isolation and Validation by RT-qPCR

Based on the microarray analysis results, four hub genes were screened out for further validation using RT-qPCR in an independent patient cohort (Table 2). First, we collected blood samples, and then we isolated the peripheral blood mononuclear cells (PBMCs). Total RNAs were extracted from the PBMCs using TRIzol reagent (Takara, Kyoto, Japan), then the TransScript^®^First-Strand cDNA Synthesis SuperMix (Transgen, Beijing, China) was used to synthesize complementary DNA (cDNA), referring to the instructions of the manufacturer. RT-qPCR was utilized with a Step One Plus real-time PCR system (Applied Biosystems, Los Angeles, CA, United States) using the SYBR^®^ Premix Ex Taq^TM^ (Takara, Kyoto, Japan). We used the 2^–ΔΔ*Ct*^ method to quantify gene expression. Primers for the selected four hub genes were designed by Sangon Biotech (Shanghai, China), and their sequences are available in Table 2. We used reference 18s ribosomal RNA to normalize the expression data (Wang et al., 2019).

**TABLE 2:** PCR primers for quantitative real-time PCR.

Gene	Primer sequence (5′→3′)	
CD40	F: CACAGCTCGAAGAGTGGTGA	R: ATGGGAAAGCCAAATCTCCCT
F11R	F: ATCCCCTGTCAGCCTCTGAT	R: TGCGCACAGCATTTGAAGTC
TNRC18	F: CAGCGACGACGACCTGTG	R: TTCCCTTTCTTCTTCTTCCTGGG
CAMK2G	F: TCTCCTCCTCTTGCTCCCTC	R: GACCACAGAGAAAGCACCCT
18SrRNA	F: GTAACCCGTTGAACCCCATT	R: CCATCCAATCGGTAGTAGCG

### Enzyme-Linked Immunosorbent Assay

Blood samples were collected from another 60 non-diabetic patients with ≥50% stenosis in ≥1 major vessel and 40 patients with luminal stenosis of <50%. Blood plasma samples were collected from the upper and stored at −80°C after centrifuging at 1,000 *g* for 10 min at room temperature before the ELISA. Serum concentrations of CD40, F11R, TNRC18, and CAMK2G protein were measured using ELISA kits referring to the manufacturer’s instructions (MEIMIAN, Jiangsu Meimian Industrial Co., Ltd.). Mouse monoclonal antibodies to CD40, F11R, TNRC18, and CAMK2G (MEIMIAN, Jiangsu Meimian Industrial Co., Ltd.) and the horseradish peroxidase (HRP)-conjugated secondary antibodies of rabbit immunoglobulin G (IgG, MEIMIAN, Jiangsu Meimian Industrial Co., Ltd.) were used. The optical density (OD) values of the samples were obtained using a spectrophotometer at 450 nm. The concentrations were calculated by comparing the OD values of the samples with the standard curve. All of the experiments were performed at least three times (Sun et al., 2018).

### Atherosclerosis Model and Histological Analysis

The animal study was approved by the Experimental Animal Research Committee of Weifang Medical University, and all of the animal care and experimental procedures were in accordance with the recommendations in the Guide for the Care and Use of Laboratory Animals of the National Institutes of Health. Eight-week-old ApoE^–/–^ mice were fed a common or high-fat diet for 25 weeks. The aorta tissues were harvested and fixed in 4% paraformaldehyde in phosphate-buffered saline (PBS) for histological and morphological staining. Six-micrometer-thick cryosections were obtained sequentially, beginning at the junction of the left ventricle and the aorta. The sections were stained with hematoxylin and eosin. Immunohistochemical (IHC) staining using antibodies against CD40, F11R, TNRC18, and CAMK2G (Santa Cruz Biotechnology Inc., Santa Cruz, CA, United States) was performed according to the manufacturer’s description. The slides were inspected under an optical microscope (Olympus BX61, Tokyo, Japan) at 200× and 400× magnification. For quantification analysis, the percentage of positive area for immunohistochemistry was determined using ImageJ software (National Institutes of Health, Bethesda, MD, United States) (Liu et al., 2016).

### Statistical Analysis

A Student’s *t*-test was performed to assess statistical variations between Case (1) and Case (2) samples by SPSS 20.0 statistical software (SPSS Inc., Chicago, IL, United States). Most bioinformatics analyses including WGCNA were performed in R (version 3.5.2) with default test statistics and cutoff values as specified in individual method sections. *P* < 0.05 was considered statistically significant. Results are expressed as mean ± SEM. A *P*-value < 0.05 was considered statistically significant.

## Results

### Microarray Data Acquisition and Analysis of Gene Expression

**FIGURE 2:**
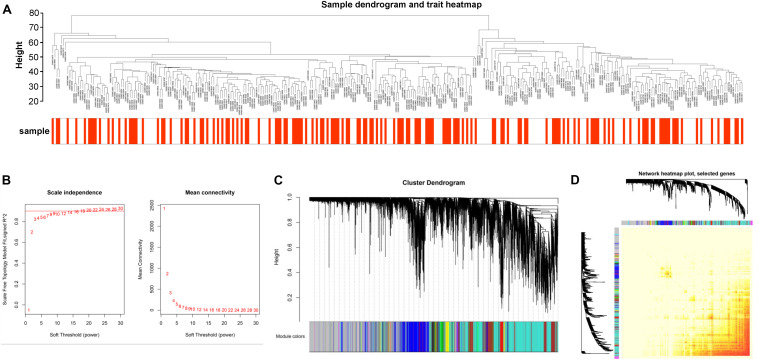
**(A)** Sample clustering to detect outliers. A cluster analysis of samples with coronary artery disease (CAD) in this study. Weighted gene co-expression network analysis (WGCNA) and flashClust were performed in the cluster analysis of 322 samples. We obtained two clusters. **(B)** Network topology analysis for adjacency matrix with a set of soft-thresholding powers. The red numbers in the drawing indicate soft-thresholding power in keeping with the correlation coefficient value. **(C)** Clustering dendrograms of genes. On the basis of topological overlap, 18 co-expression modules of genes that are associated with CAD were constructed with distinctive colors. **(D)** Visualizing the gene network by using a heat map plot. The high brightness indicates lower overlap, and the comparatively darker red color shows higher overlap.

In our study, our aim was to gain more insight into the latent molecular mechanisms of CAD in non-diabetic patients. We set “Coronary artery disease in non-diabetic patients” and “Gene expression” as the keywords to search literature and acquired the datasets GSE20680 and GSE20681. In addition, after excluding the diabetic patients, we obtained 322 samples for further WGCNA analysis, including 170 samples in Case (1) and 152 samples in Case (2). The expression profiles of 12,020 genes were gained from those 322 samples after data preprocessing (Supplementary Table S1). Mann–Whitney test analysis was used to analyze the DEGs between the Case (1) and Case (2) samples, and a total of 1,225 DEGs were identified. Then, we performed the cluster analysis using flash Clust tool package of WGCNA algorithm. In the study, the sample quality of the discovery dataset was assessed by sample clustering. A hierarchical clustering analysis was obtained, and the results are exhibited in Figure 2A. Based on this result, no samples were eliminated from the present study. And we found that all of these 322 samples were classified into two clusters. Cluster I contained 198 samples, while the other 124 samples were included in cluster II.

### Construction of Gene Co-expression Modules

We constructed the co-expression modules using the 12,020 genes in the 322 samples by the WGCNA tool package. Before constructing the weighted gene co-expression modules, we selected an appropriate power value for relative, which mainly balanced the scale independence and means connectivity of the gene module. We found that the scale independence was >0.85 and had a higher mean connectivity when the power value was set at 7 (Figure 2B). Thus, the power value was set at 7 in the following analysis. As a result, we got a hierarchical clustering tree and an aggregate of 18 co-expression modules was screened out (Figure 2C). Then, we counted the number of genes in each module. Finally, those modules were ranked from high to low based on the number of genes, which were marked with different colors.

### Intermodular Connectivity Analysis of Co-expression

We analyzed the independence among the 18 co-expression modules. Based on the TOM, we used all of the 12,020 genes to analyze the heat map (Figure 2D). The result showed that a high brightness indicated less overlap, and a darker red color represented more overlap. Moreover, there was no significant difference among the 18 co-expression modules, except for some areas with high brightness, which demonstrated that these modules had a higher degree of gene co-expression independence.

### Quantification of Module-Clinic Trait Associations

**FIGURE 3:**
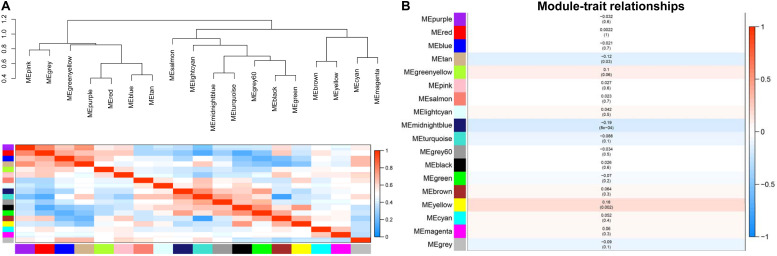
**(A)** The gene dendrogram and eigengene adjacency heat map. We obtained two clusters, including eight clusters. In the eigengene adjacency heat map, the slope of the variance in color from purple to yellow represents the connectedness of genes for various modules from strong to weak. **(B)** Module–trait relationships. The right *y*-axis represents the correlation of module significance ranked from –1 to 1. Each row covers the corresponding correlation coefficient and *P*-value.

We used an eigengene dendrogram and eigengene adjacency heat map to quantify the module clinical traits (Figure 3). First, we analyzed the connectivity and conducted cluster analysis among the 18 co-expression modules (Figure 3A). For the purpose of analyzing the degree of association between the eigengene and clinic trait and to find the significance of genes in their respective modules, gene significance (GS) was identified as the correlation of genes with CAD. Next, the mean GS of all of the genes in the module was counted and was regarded as MS. The association between each module and CAD is described in Figure 3B, and from the picture, we could determine that the midnight blue module (*r* = −0.19, *p* = 8e-4), the yellow module (*r* = 0.18, *p* = 0.002), and the tan module (*r* = −0.12, *p* = 0.03) were the modules with association with CAD in the module–feature relationship. We only chose the yellow module in which genes having the strongest positive correlation and the midnight blue module with genes having the strongest negative correlation with the disease status for further analysis, although the tan module was also positively correlated with the disease and the *P*-value < 0.05.

### Functional Enrichment Analysis of the Gene Modules

**FIGURE 4:**
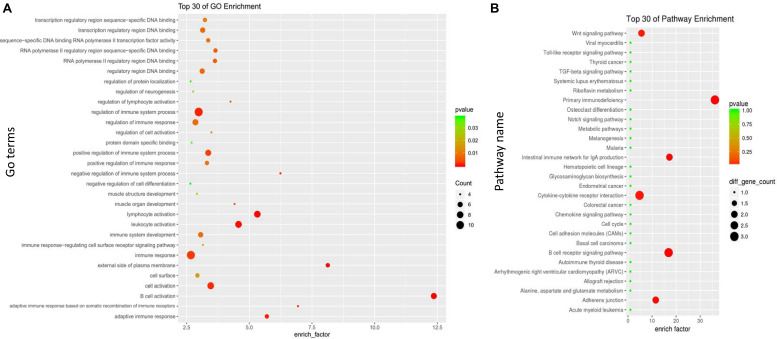
Gene Ontology (GO) and Kyoto Encyclopedia of Genes and Genomes (KEGG) enrichment analyses for the midnight blue module. **(A)** GO enrichment for the midnight blue module. The horizontal axis represents the number of genes, and the vertical axis indicates the GO terms of the module. The circle pillars represent the biological process category, the triangle pillars represent the cellular component category, and the square pillars represent the molecular function category. **(B)** Scatterplot of the enriched KEGG pathways. The enrichment element represents the proportion of the genes that amount to the total number of genes in a certain pathway. The color and size of the dots indicate the scope of *P* values and the gene amount.

**FIGURE 5:**
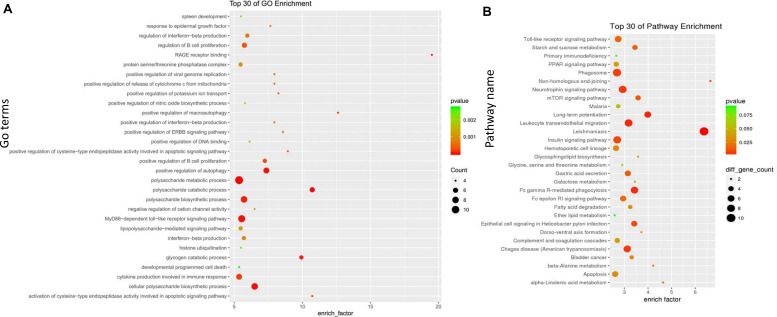
Gene Ontology (GO) and Kyoto Encyclopedia of Genes and Genomes (KEGG) enrichment analyses for the yellow module. **(A)** GO enrichment for the yellow module. The horizontal axis represents the number of genes, and the vertical axis indicates the GO terms. The circle pillars represent the biological process category, the triangle pillars represent the cellular component category, and the square pillars represent the molecular function category. **(B)** Scatterplot for enriched KEGG pathways. The enrichment element represents the proportion of the genes that amount to the total gene in a certain pathway. The color and size of the dots indicate the scope of *P* values and the gene amount.

A GO enrichment analysis was performed on the midnight blue module and the yellow module. In the midnight blue module, the GO terms of (BPs) were enriched in the regulation of immune system processes, lymphocyte activation, leukocyte activation, and the immune response. GO terms were enriched on the external side of the plasma membrane and cell surface in the CC category. In the MF category, the GO terms were mainly enriched in the transcription regulatory region for DNA binding (Figure 4A and Supplementary Table S2). In the yellow module, the GO terms of BP were enriched in the polysaccharide metabolic process, MyD88-dependent toll-like receptor signaling pathway, the production of cytokines that are involved in the immune response, and the regulation of B cell proliferation. In the CC category, the GO terms were enriched in protein serine/threonine phosphatase complex. In the MF category, the GO terms were mainly enriched in RAGE receptor binding (Figure 5A and Supplementary Table S3).

Referring to the KEGG databases, a pathway annotation of genes in the midnight blue module and the yellow module was performed, and we finally acquired the interrelated pathway terms. In the midnight blue module, the genes were mainly enriched in the Wnt signaling pathway, primary immunodeficiency, intestinal immune network for IgA production, B cell receptor signaling pathway, and adherens junctions (Figure 4B and Supplementary Table S4). In the yellow module, the pathway terms were mainly enriched in phagosomes, neurotrophin signaling pathway, leukocyte transendothelial migration, leishmaniasis, etc. (Figure 5B and Supplementary Table S5).

In summary, we identified the midnight blue and the yellow modules for further analysis, and we found that the genes in the two modules displayed a high association with the immuno-inflammatory response.

### Identification of Hub Genes Associated With the Pathogenesis of Coronary Artery Disease

**FIGURE 6:**
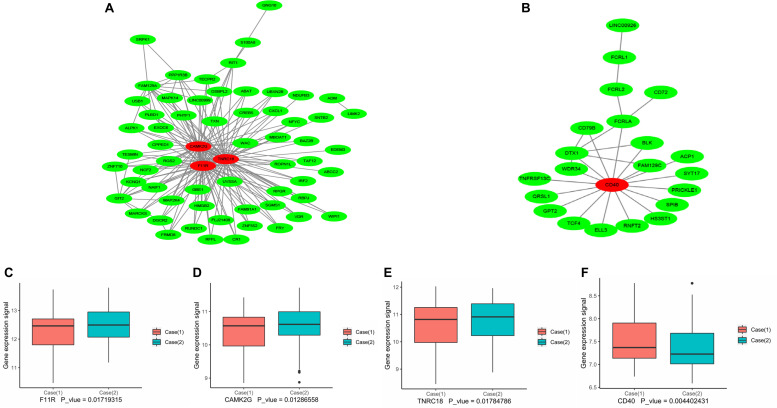
Protein–protein interaction network analysis using Cytoscape software. The results of the network analysis for dramatically changed proteins. A co-expression network of genes in the yellow module **(A)** and the midnight blue module **(B)** was constructed, and the red circles represent the hub genes with the highest module membership, and the edges between two nodes indicate the connection between the two genes. **(C–F)** Expression of hub genes F11R, CAMK2G, TNRC18, CD40 in the Case (1) and Case (2) samples. The relative expression of F11R **(C)**, CAMK2G **(D)**, TNRC18 **(E)**, and CD40 **(F)** in the Case (1) and Case (2) samples from consolidated data set; Mann–Whitney test analysis was performed.

The above results revealed that the pathogenesis of CAD was closely related to the immuno-inflammatory response. To screen out the hub genes in the midnight blue module and the yellow module (Supplementary Table S1), we conducted gene network analysis using the results of WGCNA analysis with the Cytoscape bioinformatics resource platform. Finally, the hub genes F11R, TNRC18, and CAMK2G were identified in the yellow module (Figure 6A and Table 3). CD40 was selected from the midnight blue module (Figure 6B and Table 3). Moreover, we obtained similar results by using another common database for PPI network analysis – the STRING Database (data not shown), which further supported our hub gene data. In order to validate the identified hub genes, consolidated clinical samples of Case (1) and Case (2) were analyzed using WGCNA analysis. The results showed that F11R, CAMK2G, and TNRC18 was expressed higher in Case (2) samples than in Case (1) samples; otherwise, the expression of CD40 was expressed lower in Case (2) than in Case (1) samples (Figures 6C–F). Therefore, we speculated that these four hub genes might play critical roles when CAD occurred.

**TABLE 3:** Four hub genes identified in midnightblue and yellow module.

Module	Gene symbol	Description	*P* value
Yellow	CAMK2G	calcium/calmodulin dependent protein kinase II gamma	0.012865583
Yellow	F11R	F11 receptor	0.017193146
Yellow	TNRC18	trinucleotide repeat containing 18	0.017847857
Midnightblue	CD40	CD40 molecule	0.004402431

### Validation of Hub Genes With RT-qPCR, ELISA, and Immunohistochemistry

**FIGURE 7:**
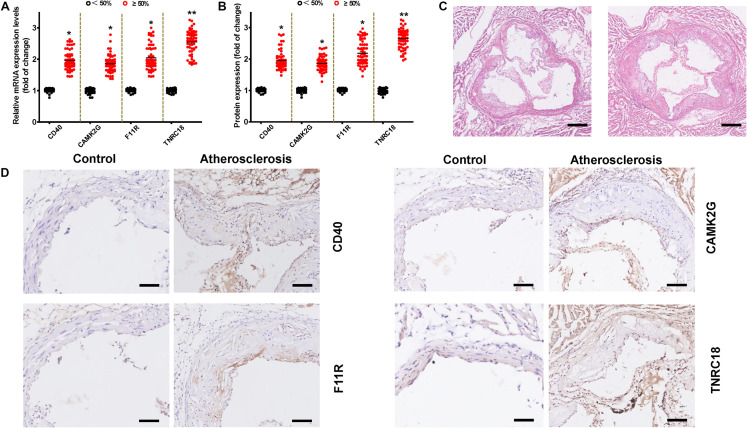
Expression levels for hub genes in validation cohort by qRT–PCR, ELISA, and immunohistochemistry (IHC). **(A)** The mRNA levels of CD40, CAMK2G, F11R, TNRC18 are demonstrated. The data are shown as the relative fold alterations in expression. **(B)** The serum levels of CD40, CAMK2G, F11R, and TNRC18 proteins for the two groups are shown. Means ± SEM, *n* = 40 in Case (1) and *n* = 60 in Case (2) groups (*P < 0.05; **P < 0.01, *t*-test). **(C)** Representative cross sections of hematoxylin and eosin staining of the aortic valve of the ApoE^– /–^ mice from each group (bar, 100 mm). **(D)** Representative CD40, F11R, TNRC18, and CAMK2G IHC staining images of the aortic valve (bar, 100 mm). The results shown are from one experiment (control group, *n* = 15; atherosclerosis group, *n* = 15).

To confirm the main conclusions that were derived from the microarray analysis, RT-qPCR was conducted. We found that most results of the RT-qPCR were qualitatively compliant with the microarray results except for CD40 (Figure 7A).

In addition, the levels of CD40, F11R, TNRC18, and CAMK2G protein products in human serum samples were analyzed by ELISA. As shown in Figure 7B, consistent with the qRT–PCR results, the total CD40, F11R, TNRC18, and CAMK2G protein levels were found increased obviously in patients with luminal stenosis of ≥50% in ≥1 major vessel compared to patients with luminal stenosis of <50%. Specially, the serum concentration of TNRC18 protein was significantly increased in patients with ≥50% stenosis in ≥1 major vessel. To further validate this conclusion, we constructed an atherosclerosis mouse model by feeding ApoE^–/–^ mice with a high-fat diet for 25 weeks. Then, we conducted hematoxylin and eosin staining (Figure 7C) and an IHC staining experiment. We found increased expression of CD40, F11R, TNRC18, and CAMK2G in atherosclerotic lesions at the junction of the left ventricle and the aorta (Figure 7D).

## Discussion

Coronary artery disease is a serious threat to human life. Although the appropriate thromboprophylaxis treatments for the patients of CAD have reduced the morbidity and mortality to some extent (Lau and Lip, 2014), effective therapeutic targets to prevent and treat CAD need to be further developed. In our study, WGCNA was used to analyze the interactions among the eigengenes of CAD by constructing a gene co-expression module that was relevant to the occurrence and development of CAD. A total of 18 co-expression modules were identified by WGCNA. We revealed that the major BPs when CAD occurred were the immune and inflammatory responses. The four hub genes CD40, F11R, TNRC18, and CAMK2G were screened out from the midnight blue and the yellow modules. In general statistics work under certain restrictions, the hub genes we identified here are calculated based on a fixed cutoff value, which has been described in the “Materials and Methods” section; however, it is not surprising that the results may vary when a different cutoff value is given. Therefore, we hypothesized that these four hub genes might be the diagnostic biomarkers and/or therapeutic targets for CAD, and that further study is needed to explore them in detail.

CD40 is a costimulatory molecule that is activated by the CD40 ligand and synthesized by natural killer (NK) cells, monocytes, and B lymphocytes (Lapinski et al., 2014) as a part of the inflammatory response. The expression level of CD40 is low under normal physiological conditions, but it may be significantly upregulated under pathological conditions (Chatzigeorgiou et al., 2010). The CD40/CD40L system may be responsible for antigen presentation and immune response and is significantly associated with the activation of T lymphocytes and macrophages, thereby exerting critical biological roles in different cell types by the modulation of different signaling pathways (Kuo et al., 2012; Shock et al., 2015). Recently, the role of CD40/CD40L interactions in atherothrombosis has been widely accepted (Antoniades et al., 2009; Zhang et al., 2013). The possible mechanisms by which the CD40 and CD40L systems affected atherosclerosis might be correlated with the immune inflammatory reaction (Gocmen et al., 2011; Fu et al., 2013; Hashem et al., 2014). Consistent with the results of the study by Chen et al. (2017), we also found that both CD40 mRNA and protein expression levels were increased in the patients with luminal stenosis of ≥50% in ≥1 major vessel compared with the control group, though the level of CD40 was downregulated through bioinformatics analysis. The current discrepancy may result from the following possibilities. First, the target gene expression data are different, as we described in the “Materials and Methods” section, we did not analyze the source data as they were; instead, we reintegrated the two data sets (GSE 20680 and GSE 20681) according to whether the degree of coronary artery stenosis was more than 50% on the basis of excluding diabetic patients. Second, part of the methodology of bioinformatics analysis is dissimilar. A traditional method was applied in the original study; however, we used a more advanced WGCNA method in the current study. Third, the demographic patterns, lesion severity estimation, and other affecting factors (such as medicament, diet, excise, etc.) may vary between the discovery and validation cohorts. Besides, we have to acknowledge that the small sample size in the validation cohort may lead to certain deviations for the overall prediction. Finally, bioinformatics analysis is not always in line with the validated result; therefore, we have to make the interpretation with great care and warrants validation with a large sample cohort.

F11R is a gene that encodes a protein called the F11 receptor. F11R was regarded as a critical gene in platelet activation, aggregation, and adhesion (Kornecki et al., 1990; Babinska et al., 2007). The F11R protein also goes by the name of junctional adhesion molecule (JAM-1 or JAM-A), which was identified at tight junctions of vascular endothelial and epithelial cells in 1998 (Martin-Padura et al., 1998). A recent report revealed that the mRNA and protein levels of F11R/JAM-A were increased in the atherosclerotic plaques of patients with advanced aortic and peripheral vascular disease. In addition, it played a pivotal role in inflammatory thrombosis and triggering atherosclerosis (Babinska et al., 2007). Some researchers also verified that elevated levels of F11R in the circulation of patients were correlated with the severity of CAD (Cavusoglu et al., 2007) and hypertension (Ong et al., 2009), which revealed the significant role of F11R in cardiovascular disease. Consistent with the above results, we also found that F11R mRNA and protein expression levels were all increased in the case group compared with the control group. Therefore, we proposed that therapeutic drugs that antagonized the function of F11R/JAM-A could serve as a novel strategy to prevent and treat CAD. Of course, we needed to rule out the influence of hypertension on its expression because the percentage of hypertension patients in case (2) samples we used for verification was obviously higher than that of the case (1) samples.

Trinucleotide repeat containing 18 (TNRC18) is a gene with an undetermined function, as there are presently few reports about it (Chu et al., 2017). In addition, there have been no studies referring to the expression of TNRC18 in CAD patients. Our research showed for the first time that TNRC18 exhibited a high fold increase in the case group compared with the control group. Our results demonstrated that a high expression level of TNRC18 was detected in the serum of patients with ≥50% stenosis in at least one major vessel, indicating a higher probability of immuno-inflammatory response involvement in the pathogenesis of CAD. In addition, a systematic biological analysis revealed that immune and inflammatory factors might be associated with the occurrence and development of CAD. Therefore, we proposed that TNRC18 overexpression had a functional association with the pathogenesis of CAD, and that TNRC18 might be a latent candidate biomarker for CAD diagnosis. However, further studies are needed to confirm this conclusion.

CAMK2G is the gamma isoform of CAMK2 and could catalyze the formation of a series of the second messenger of Ca^2+^. In the past decade, many studies have shown that CAMK2 was a pivotal regulator of cardiac function. For instance, CaMKII plays a critical role not only in the transcriptional activation that is associated with cardiac hypertrophy but also in apoptosis and aberrant Ca^2+^ handling, which contribute to heart failure (Zhang and Brown, 2004). One recent study reported that there was a significant association between the cAMP signaling pathway and CAMK2 *via* the regulation of phosphodiesterase 4D (Mika et al., 2015). Sag et al. (2009) found that CAMK2 was associated with electrical remodeling after myocardial infarction and arrhythmias (Mohler and Hund, 2011). Therefore, combined with our research, it might provide a specific target for further insights on the pathogenesis of CAD.

Comparing the present results with other WGCNAs in the setting of CAD, our results will provide a direction for further study in the gene regulatory mechanisms in CAD patients without diabetic status. However, further experiments with larger samples are needed to confirm the accuracy of TNRC18 in CAD diagnosis. Furthermore, in our future research, we will verify the correlation between TNRC18 level and future treatment modalities and further determine the underlying immuno-inflammatory mechanisms of TNRC18 in CAD patients without diabetic status.

## Limitations

Our study has several limitations. First, the study reanalyzed data from a GEO database and did not conduct any robust *in vivo* or *in vitro* studies for mechanistic exploration to support the conclusion. Second, in our study, we analyzed RNA from isolated mononuclear cells in contrast with the whole blood from all patients, as used in the study by Sinnaeve et al. (2009). This may be one of the causes for the limited overlap between our study and the Sinnaeve study at the individual gene level. Third, we used a series of small sample sizes to validate the gene expression in CAD patients without diabetic status. One particularly significant imbalance in our cohorts is due to the baseline hypertension level and medications. A larger, truly prospective study with less unidentified confounders should be conducted to validate this conclusion.

## Conclusion

Generally, our research explored several hub genes relevant to CAD in non-diabetic patients through WGCNA analysis, including CD40, F11R, TNRC18, and CAMK2G. Those genes, especially TNRC18, might be potential candidates as diagnostic biomarkers and/or therapeutic targets for CAD patients without diabetic status.

## Data Availability Statement

The datasets in this research are available on request to the corresponding author. The datasets GSE20680 and GSE20681 could be downloaded from the NCBI Gene Expression Omnibus (GEO) database (http://www.ncbi.nlm.nih.gov/geo/). The scripts for data preprocessing and WGCNA network construction are available at GitHub repository (https://github.com/qijianbaimo/WGCNA_coronary_artery_disease).

## Ethics Statement

The studies involving human participants were reviewed and approved by the Ethics Committee of the Affiliated Hospital of Weifang Medical University. The patients/participants provided their written informed consent to participate in this study. The animal study was reviewed and approved by the Experimental Animal Research Committee of Weifang Medical University.

## Author Contributions

MJ together with QZ conducted the experiment and collected the clinical sample. RL created and maintained the Github repository. JL, XX, PD, and CM assisted in clinical sample collection. YL, LW, WQ, and KK provided conceptual advice and critically reviewed the manuscript. HW and TW conceptually designed the research and prepared the manuscript.

## Conflict of Interest

The authors declare that the research was conducted in the absence of any commercial or financial relationships that could be construed as a potential conflict of interest.

## Footnotes

1 http://www.ncbi.nlm.nih.gov/geo

2 http://string-db.org/

3 http://apps.cytoscape.org/apps/keggscape
