# Supplementary material for: Identification of Four Potential Biomarkers Associated With Coronary Artery Disease in Non-diabetic Patients by Gene Co-expression Network Analysis
Source: Front Genet. 2020 Jun 24;11:542. doi: 10.3389/fgene.2020.00542 (PMC7344232; doi:10.3389/fgene.2020.00542)
Supplement: Supplementary file 2 [file Table_2.docx]

| **Table S2 \| Gene ontology (GO) enrichment analysis in midnightblue module** | | | | |
| --- | --- | --- | --- | --- |
| **ID** | **Category** | **Term** | **Count** | **P-value** |
| BP (biological process) | GO:0032880 | regulation of protein localization | 4 | 3.81E-02 |
|  | GO:0050767 | regulation of neurogenesis | 4 | 3.30E-02 |
|  | GO:0051249 | regulation of lymphocyte activation | 4 | 5.80E-03 |
|  | GO:0002682 | regulation of immune system process | 11 | 9.39E-04 |
|  | GO:0050776 | regulation of immune response | 7 | 7.76E-03 |
|  | GO:0050865 | regulation of cell activation | 4 | 1.32E-02 |
|  | GO:0002684 | positive regulation of immune system process | 7 | 2.87E-03 |
|  | GO:0050778 | positive regulation of immune response | 5 | 9.43E-03 |
|  | GO:0002683 | negative regulation of immune system process | 4 | 1.11E-03 |
|  | GO:0045596 | negative regulation of cell differentiation | 4 | 3.88E-02 |
|  | GO:0061061 | muscle structure development | 4 | 2.69E-02 |
|  | GO:0007517 | muscle organ development | 4 | 4.97E-03 |
|  | GO:0046649 | lymphocyte activation | 8 | 5.97E-05 |
|  | GO:0045321 | leukocyte activation | 8 | 1.88E-04 |
|  | GO:0002520 | immune system development | 6 | 8.36E-03 |
|  | GO:0002768 | immune response-regulating cell surface receptor signaling pathway | 4 | 1.97E-02 |
|  | GO:0006955 | immune response | 11 | 2.42E-03 |
|  | GO:0001775 | cell activation | 8 | 1.38E-03 |
|  | GO:0042113 | B cell activation | 7 | 3.64E-07 |
|  | GO:0002460 | adaptive immune response | 4 | 6.91E-04 |
|  | GO:0002250 | adaptive immune response | 5 | 6.28E-04 |
| MF (molecular function) | GO:0000976 | transcription regulatory region sequence-specific DNA binding | 5 | 1.05E-02 |
|  | GO:0044212 | transcription regulatory region DNA binding | 6 | 7.27E-03 |
|  | GO:0000981 | sequence-specific DNA binding RNA polymerase II transcription factor activity | 5 | 8.74E-03 |
|  | GO:0000977 | RNA polymerase II regulatory region sequence-specific DNA binding | 5 | 5.89E-03 |
|  | GO:0001012 | RNA polymerase II regulatory region DNA binding | 5 | 6.04E-03 |
|  | GO:0000975 | regulatory region DNA binding | 6 | 7.47E-03 |
|  | GO:0019904 | protein domain specific binding | 4 | 3.60E-02 |
| CC (cellular component) | GO:0009897 | external side of plasma membrane | 5 | 9.50E-05 |
|  | GO:0009986 | cell surface | 5 | 1.65E-02 |
