# Supplementary material for: Identification of Four Potential Biomarkers Associated With Coronary Artery Disease in Non-diabetic Patients by Gene Co-expression Network Analysis
Source: Front Genet. 2020 Jun 24;11:542. doi: 10.3389/fgene.2020.00542 (PMC7344232; doi:10.3389/fgene.2020.00542)
Supplement: Supplementary file 3 [file Table_3.docx]

| **Table S3 \| Gene ontology (GO) enrichment analysis in yellow module** | | | | |
| --- | --- | --- | --- | --- |
| **ID** | **Category** | **Term** | **Count** | **P-value** |
| BP (biological process) | GO:0048536 | spleen development | 4 | 0.00246 |
|  | GO:0070849 | response to epidermal growth factor | 4 | 0.00064 |
|  | GO:0032648 | regulation of interferon-beta production | 5 | 0.00067 |
|  | GO:0030888 | regulation of B cell proliferation | 6 | 0.00031 |
|  | GO:0045070 | positive regulation of viral genome replication | 4 | 0.00055 |
|  | GO:0090200 | positive regulation of release of cytochrome c from mitochondria | 4 | 0.00055 |
|  | GO:0043268 | positive regulation of potassium ion transport | 4 | 0.00047 |
|  | GO:0045429 | positive regulation of nitric oxide biosynthetic process | 4 | 0.00199 |
|  | GO:0016239 | positive regulation of macroautophagy | 4 | 8.3E-05 |
|  | GO:0032728 | positive regulation of interferon-beta production | 4 | 0.00055 |
|  | GO:1901186 | positive regulation of ERBB signaling pathway | 4 | 0.0004 |
|  | GO:0043388 | positive regulation of DNA binding | 4 | 0.00158 |
|  | GO:0006919 | activation of cysteine-type endopeptidase activity involved in apoptotic process | 7 | 0.00098 |
|  | GO:0030890 | positive regulation of B cell proliferation | 5 | 0.00026 |
|  | GO:0010508 | positive regulation of autophagy | 7 | 2.4E-05 |
|  | GO:0005976 | polysaccharide metabolic process | 11 | 5.7E-06 |
|  | GO:0000272 | polysaccharide catabolic process | 6 | 8.7E-06 |
|  | GO:0000271 | polysaccharide biosynthetic process | 8 | 4.9E-05 |
|  | GO:2001258 | negative regulation of cation channel activity | 4 | 0.00125 |
|  | GO:0002755 | MyD88-dependent toll-like receptor signaling pathway | 9 | 2.5E-05 |
|  | GO:0031663 | lipopolysaccharide-mediated signaling pathway | 5 | 0.00101 |
|  | GO:0032608 | interferon-beta production | 5 | 0.00083 |
|  | GO:0016574 | histone ubiquitination | 4 | 0.00246 |
|  | GO:0005980 | glycogen catabolic process | 5 | 5.4E-05 |
|  | GO:0010623 | developmental programmed cell death | 4 | 0.00273 |
|  | GO:0002367 | cytokine production involved in immune response | 7 | 0.00019 |
|  | GO:0033692 | cellular polysaccharide biosynthetic process | 8 | 1.9E-05 |
|  | GO:0006919 | activation of cysteine-type endopeptidase activity involved in apoptotic process | 7 | 0.00098 |
| MF (molecular function) | GO:0050786 | RAGE receptor binding | 4 | 1.5E-05 |
| CC (cellular component) | GO:0008287 | protein serine/threonine phosphatase complex | 5 | 0.00101 |
