# Supplementary material for: Identification of Four Potential Biomarkers Associated With Coronary Artery Disease in Non-diabetic Patients by Gene Co-expression Network Analysis
Source: Front Genet. 2020 Jun 24;11:542. doi: 10.3389/fgene.2020.00542 (PMC7344232; doi:10.3389/fgene.2020.00542)
Supplement: Supplementary file 4 [file Table_4.docx]

| **Table S4 \| KEGG enrichment analysis of genes in midnightblue module** | | | | |
| --- | --- | --- | --- | --- |
| **ID** | **Category** | **Term** | **Count** | **P-value** |
| KEGG_PATHWAY | hsa04310 | Wnt signaling pathway | 2 | 1.63E-02 |
|  | hsa05416 | Viral myocarditis | 1 | 1 |
|  | hsa04620 | Toll-like receptor signaling pathway | 1 | 1 |
|  | hsa05216 | Thyroid cancer | 1 | 1 |
|  | hsa04350 | TGF-beta signaling pathway | 1 | 1 |
|  | hsa05322 | Systemic lupus erythematosus | 1 | 1 |
|  | hsa00740 | Riboflavin metabolism | 1 | 1 |
|  | hsa05340 | Primary immunodeficiency | 3 | 2.37E-04 |
|  | hsa04380 | Osteoclast differentiation | 1 | 1 |
|  | hsa04330 | Notch signaling pathway | 1 | 1 |
|  | hsa01100 | Metabolic pathways | 1 | 1 |
|  | hsa04916 | Melanogenesis | 1 | 1 |
|  | hsa05144 | Malaria | 1 | 1 |
|  | hsa04672 | Intestinal immune network for IgA production | 2 | 6.99E-04 |
|  | hsa04640 | Hematopoietic cell lineage | 1 | 1 |
|  | hsa00534 | Glycosaminoglycan biosynthesis | 1 | 1 |
|  | hsa05213 | Endometrial cancer | 1 | 1 |
|  | hsa04060 | Cytokine-cytokine receptor interaction | 3 | 1.29E-02 |
|  | hsa05210 | Colorectal cancer | 1 | 1 |
|  | hsa04062 | Chemokine signaling pathway | 1 | 1 |
|  | hsa04110 | Cell cycle | 1 | 1 |
|  | hsa04514 | Cell adhesion molecules (CAMs) | 1 | 1 |
|  | hsa05217 | Basal cell carcinoma | 1 | 1 |
|  | hsa04662 | B cell receptor signaling pathway | 3 | 1.33E-04 |
|  | hsa05320 | Autoimmune thyroid disease | 1 | 1 |
|  | hsa05412 | Arrhythmogenic right ventricular cardiomyopathy (ARVC) | 1 | 1 |
|  | hsa05330 | Allograft rejection | 1 | 1 |
|  | hsa00250 | Alanine, aspartate and glutamate metabolism | 1 | 1 |
|  | hsa04520 | Adherens junction | 2 | 2.19E-03 |
|  | hsa05221 | Acute myeloid leukemia | 1 | 1 |
