# Supplementary material for: Identification of Four Potential Biomarkers Associated With Coronary Artery Disease in Non-diabetic Patients by Gene Co-expression Network Analysis
Source: Front Genet. 2020 Jun 24;11:542. doi: 10.3389/fgene.2020.00542 (PMC7344232; doi:10.3389/fgene.2020.00542)
Supplement: Supplementary file 5 [file Table_5.docx]

| **Table S5 \| KEGG enrichment analysis of genes in yellow module** | | | | |
| --- | --- | --- | --- | --- |
| **ID** | **Category** | **Term** | **Count** | **P-value** |
| KEGG_PATHWAY | hsa04620 | Toll-like receptor signaling pathway | 6 | 1.66E-02 |
|  | hsa00500 | Starch and sucrose metabolism | 4 | 1.48E-02 |
|  | hsa05340 | Primary immunodeficiency | 2 | 8.47E-02 |
|  | hsa03320 | PPAR signaling pathway | 4 | 4.05E-02 |
|  | hsa04145 | Phagosome | 9 | 6.13E-03 |
|  | hsa03450 | Non-homologous end-joining | 2 | 7.61E-03 |
|  | hsa04722 | Neurotrophin signaling pathway | 8 | 5.02E-03 |
|  | hsa04150 | mTOR signaling pathway | 4 | 1.27E-02 |
|  | hsa05144 | Malaria | 3 | 5.38E-02 |
|  | hsa04720 | Long-term potentiation | 6 | 2.20E-03 |
|  | hsa04670 | Leukocyte transendothelial migration | 8 | 2.93E-03 |
|  | hsa05140 | Leishmaniasis | 10 | 2.54E-06 |
|  | hsa04910 | Insulin signaling pathway | 8 | 8.53E-03 |
|  | hsa04640 | Hematopoietic cell lineage | 5 | 2.86E-02 |
|  | hsa00601 | Glycosphingolipid biosynthesis | 2 | 4.01E-02 |
|  | hsa00260 | Glycine, serine and threonine metabolism | 2 | 6.79E-02 |
|  | hsa04971 | Gastric acid secretion | 5 | 1.31E-02 |
|  | hsa00052 | Galactose metabolism | 2 | 4.42E-02 |
|  | hsa04666 | Fc gamma R-mediated phagocytosis | 7 | 2.99E-03 |
|  | hsa04664 | Fc epsilon RI signaling pathway | 5 | 1.77E-02 |
|  | hsa00071 | Fatty acid degradation | 3 | 3.12E-02 |
|  | hsa00565 | Ether lipid metabolism | 2 | 9.07E-02 |
|  | hsa05120 | Epithelial cell signaling in Helicobacter pylori infection | 5 | 8.82E-03 |
|  | hsa04320 | Dorso-ventral axis formation | 2 | 3.62E-02 |
|  | hsa04610 | Complement and coagulation cascades | 4 | 3.84E-02 |
|  | hsa05142 | Chagas disease (American trypanosomiasis) | 7 | 5.17E-03 |
|  | hsa05219 | Bladder cancer | 3 | 2.89E-02 |
|  | hsa00410 | beta-Alanine metabolism | 2 | 2.59E-02 |
|  | hsa04210 | Apoptosis | 5 | 3.00E-02 |
|  | hsa00592 | alpha-Linolenic acid metabolism | 2 | 2.01E-02 |
